# SuRVoS 2: Accelerating Annotation and Segmentation for Large Volumetric Bioimage Workflows Across Modalities and Scales

**DOI:** 10.3389/fcell.2022.842342

**Published:** 2022-04-01

**Authors:** Avery Pennington, Oliver N. F. King, Win Min Tun, Elaine M. L. Ho, Imanol Luengo, Michele C. Darrow, Mark Basham

**Affiliations:** ^1^ Diamond Light Source Ltd., Didcot, United Kingdom; ^2^ The Rosalind Franklin Institute, Didcot, United Kingdom

**Keywords:** segmentation (image processing), annotation, U-net, volume electron microscopy (vEM), X-ray microscopy imaging, open source software, python (programming language), computer vision

## Abstract

As sample preparation and imaging techniques have expanded and improved to include a variety of options for larger sized and numbers of samples, the bottleneck in volumetric imaging is now data analysis. Annotation and segmentation are both common, yet difficult, data analysis tasks which are required to bring meaning to the volumetric data. The SuRVoS application has been updated and redesigned to provide access to both manual and machine learning-based segmentation and annotation techniques, including support for crowd sourced data. Combining adjacent, similar voxels (supervoxels) provides a mechanism for speeding up segmentation both in the painting of annotation and by training a segmentation model on a small amount of annotation. The support for layers allows multiple datasets to be viewed and annotated together which, for example, enables the use of correlative data (e.g. crowd-sourced annotations or secondary imaging techniques) to guide segmentation. The ability to work with larger data on high-performance servers with GPUs has been added through a client-server architecture and the Pytorch-based image processing and segmentation server is flexible and extensible, and allows the implementation of deep learning-based segmentation modules. The client side has been built around Napari allowing integration of SuRVoS into an ecosystem for open-source image analysis while the server side has been built with cloud computing and extensibility through plugins in mind. Together these improvements to SuRVoS provide a platform for accelerating the annotation and segmentation of volumetric and correlative imaging data across modalities and scales.

## Introduction

The volume electron microscopy (vEM) and X-ray imaging ecosystems have flourished in recent years, through improvements to previously used techniques and the development of new techniques, all providing functional understandings through structural study (Peddie and Collinson 2014; Yoshiyuki et al., 2018; Xu et al., 2021). It is now common practice to collect 100s of GB of data daily across multiple correlative modalities contributing to the same project. This has shifted the experimental bottleneck to the image processing and analysis pipeline. Often, functional insights can only come from detailed segmentation and annotation of the image data, which currently is completed manually by an expert researcher. This creates a significant probability of creating “Dark Data,” data that is collected, but due to time constraints and complexity, is not fully analyzed, or unintentionally analyzed in a biased way. Of specific concern are “Self Selection” and “Summaries of Data” (Hand, 2020), which are both routinely used to reduce the analytical load on the researcher. Our anecdotal experience supports this and shows that the vast majority of the data that is currently collected goes unanalyzed, or is only partially analyzed, and in many cases the analysis that is performed is qualitative or only takes into consideration a small number of examples or features due to the onerous nature of the task.

There are general computational tools available to ease some of the burden of the segmentation and image analysis process such as IMOD (Kremer et al., 1996), Ilastik (Berg et al., 2017), ImageJ (Abràmoff et al., 2004) and its many plugins, such as Weka (Hall et al., 2009), or commercial software such as Avizo (ThermoFisher.com, 2021). In addition, certain fields such as neuroanatomy have specialized image analysis and segmentation tools such as Knossos (Helmstaedter et al., 2011) that can perform some of these tasks. However, all these tools still often require specialized technical knowledge, substantial experimentation, and often scripting to adapt to complex data, especially data with artefacts or low signal-to-noise ratio. In all cases where machine learning is used, capturing a dataset of expert segmentation or annotations is a necessary first step to explore which methodologies provide the best results. This process works well if the team is composed of both biological and data science experts, however, there is still a significant challenge in providing sufficient amounts of high-quality annotation in a timely fashion.

Recently, several tools that allow non-specialists to use deep learning for image segmentation have been developed. CellProfiler 3.0 combines both a wizard-like interface for applying U-Net models and an extensive suite of tools for shallow machine learning-based segmentation and for analysis of the resulting segmentation (McQuin et al., 2018). CDeep3M is a cloud-based solution for applying deep learning to segmentation but has a command line interface (Haberl et al., 2018). DeepMIB provides a wizard-like interface for running a deep-learning pipeline but doesn’t provide any support for developing annotation or analyzing the resulting segmentation (Belevich and Jokitalo, 2021). DeepImageJ, which, in conjunction with ImageJ and Fiji itself, provides a pipeline for prediction of U-Net-based segmentation models as well as many tools for the preparation of training data and the analysis of results (Gomez-de-Mariscal et al., 2021). SuRVoS2 is unique in providing a self-contained GUI tool designed for non-programmers that allows painting of annotations using super-regions, a machine-learning based prediction mechanism for accelerating the annotation process, an integrated system for training and prediction of a U-net model, and tools for analyzing the resulting segmentation.

**FIGURE 1:**
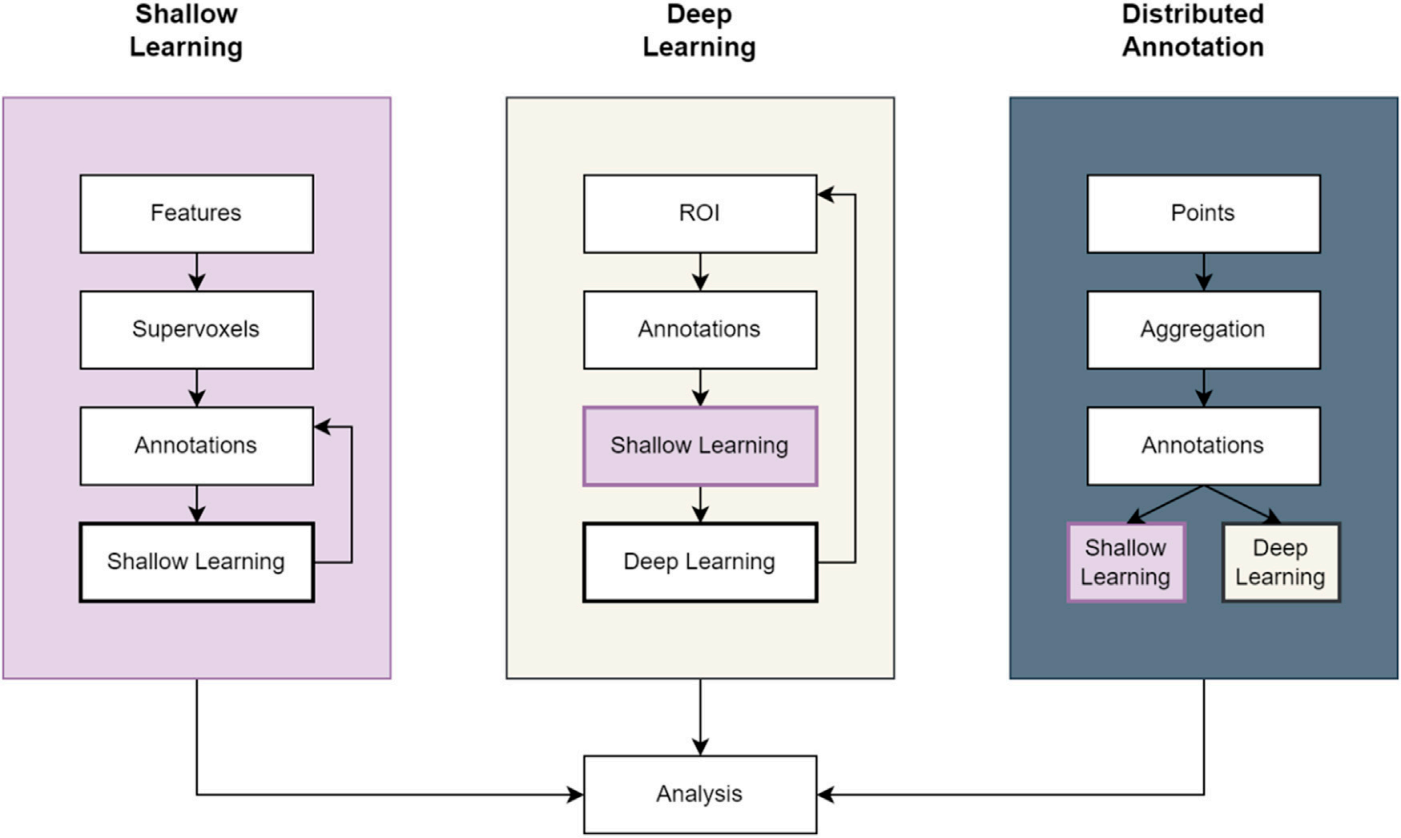
Example workflow pipelines in SuRVoS2. The Shallow Learning pipeline is the same as previously available in SuRVoS using an iterative painting and predicting cycle to produce an output segmentation. The Deep Learning pipeline incorporates the Shallow Learning pipeline to quickly generate expert segmentations on a region of interest (ROI) which can subsequently be used to train a deep learning model for application to the full volume. And finally, the Distributed Annotation pipeline uses geometric data alongside either the Shallow Learning or Deep Learning pipelines to segment objects marked by 3D points.

SuRVoS Workbench (Darrow et al., 2017; Luengo et al., 2017) was originally developed to address this need for an accelerated process of producing initial expert segmentation on which to base subsequent machine learning methods. Annotation in SuRVoS is based around the concept of supervoxels (Lucchi, et al., 2011) which provide a way to select a large number of voxels in 3D with little user effort, yet still respect the boundaries found within the data itself. After using supervoxels to quickly annotate regions within a volume, an iterative shallow machine learning strategy with integrated filters for data augmentation could be applied to predict the label assignments of the volume (Figure 1). This involves pre-calculating image features and training a machine learning method such as random forests or SVM (Support Vector Machine) on the provided scribble annotation, and then to predict the segmentation of the whole volume. Because the image features are calculated separately from the machine learning algorithm, this method is considered a “shallow” machine learning approach. A novel hierarchical strategy could restrict annotations and predictions based on parent-child relationships and a “label-splitter” functionality could be used to separate out objects based on their inherent properties for analysis.

Overall, SuRVoS provided a processing pipeline which sped up the segmentation process, in some cases enabling research which wouldn’t have been possible if segmentation was completed manually (Strotton et al., 2018). However, with increasing data sizes and rates of collection, SuRVoS became cumbersome due to local memory requirements. We also received multiple requests for usability improvements, especially around installation on various operating systems. And finally, our research and implementation of deep machine learning strategies has advanced. Together, these reasons have motivated the release of SuRVoS2 as both an API backend server and a new client based on the Napari (Napari Contributors, 2021) ecosystem. This edition includes a new client-server architecture, integration of Dask (Rocklin, 2015) for parallelized image processing, and both semi-automatic shallow and deep machine learning pipelines.

## Methods

Segmentation of large 3D volumetric imaging data presents challenges in providing computing resources, project management, and in visualization and interactivity. SuRVoS2 (licensed under Apache 2.0; Basham, 2021) approaches these challenges with the ethos of providing open-source, free-to-use options for accelerated annotation, segmentation and analysis that are data type agnostic.

### Client-Server Architecture

Volumetric image segmentation is computationally intensive, but by way of its implementation as a client-server application, SuRVoS2 can be run on high-performance servers (e.g. multi-processor, multi-GPU, or high random access memory (RAM) machines) yet provide an interactive experience for users. The server can be used to run compute-heavy operations, such as segmentation algorithms, while the client is run on a convenient device local to the user, such as a laptop. The client connects to the server and directs the segmentation workflow, and all the intermediate files are stored on a server-accessible file system, with the client downloading only the data needed for interactive segmentation and visualization.

The client uses a REST (Representational State Transfer) API implemented with the Hug Library (Crosley, 2016) to make requests to the SuRVoS2 server. The REST API uses HTTP (Hypertext Transfer Protocol) to implement a clean interface for functionality such as generating features, segmentation, and analysis. SuRVoS2 can be run headless on a cluster or virtual machine and then the Napari-based client connects over HTTP. This allows the server to run as a service on HPC-based clusters, for example, and could be containerized using Docker (Merkel, 2014) in order to deploy it at large scale in one of the major cloud providers. This enables the core functionality of SuRVoS2 to be run on appropriate computational resources with fast access to the image data resulting in a faster process for the user and enabling the processing of much larger datasets, limited only by server RAM. Additionally, the integration of the REST API and separating the core logic from the user interface allows for future extension of the client side to other devices or browser-based clients, which would further increase access to SuRVoS2 tools and functionality.

### Workspace Philosophy

The SuRVoS2 server gathers and manages the information required throughout a complex segmentation project through the use of “Workspaces.” A workspace is created from an imaging volume and as the segmentation workflow of choice is used (Figure 1), all objects created (features, annotations, supervoxels, segmentation pipelines and analysis outputs; *See* Table 1) and the state of the workflow are stored in the workspace file system. The parameters used throughout the workflow can be saved and loaded into a new workspace for application to a naïve dataset. The data objects are generally stored as chunked HDF5 (Hierarchical Data Format 5) files and manipulated using the h5py library (Colette et al., 2017). This chunking mechanism can be globally tuned, allowing the file access performance to be optimized for a given set of hardware.

**TABLE 1:** List of all filters/features, shallow and deep learning, and label splitter/analysis options within SuRVoS2.

Filters and features	Machine learning	Label splitter/Analysis
Produce output images for use in shallow or deep learning pipelines	Uses manual or shallow learning created training annotations to predict classes for other voxels	Inherent characteristics of data or segmented objects used to separate objects into groups
Basic Features:	Shallow Learning:	Mean Intensity
Simple Invert	Random Forest	Standard Deviation of Intensity
Invert Threshold	Extra Random Forest	Variation of Intensity
Threshold	Gradient Boosting	Volume
Rescale	Support Vector Machine (SVM)	Bounding Box Volume
Gamma Correct	Active Contour without Edges (ACWE)	Log Bounding Box Volume
Blob:	Watershed	Position X
Structure Tensor Determinant	Deep Learning:	Position Y
Frangi	2D U-Net	Position Z
Hessian Eigenvalues	3D U-Net ^a^	Bounding Box Depth
Denoising:	FPN ^a^	Bounding Box Height
Total Variation Denoise	—	Bounding Box Width
Gaussian Blur	—	Oriented Bounding Box Volume
Median	—	Log Oriented Bounding Box Volume
Wavelet	—	Oriented Bounding Box Depth
Edges:	—	Oriented Bounding Box Height
Spatial Gradient 3D	—	Oriented Bounding Box Width
Difference of Gaussians	—	—
Laplacian	—	—
Morphology:	—	—
Dilation	—	—
Erosion	—	—
Closing	—	—
Euclidean Distance Transform	—	—
Skeletonize	—	—
Neighborhood:	—	—
Gaussian Norm	—	—
Gaussian Centre	—	—

a Indicates options where training is currently done using the SuRVoS2 API externally to the graphical user interface (GUI), with the deep learning module for prediction available within the SuRVoS2 GUI.

### Napari as a Graphical User Interface (GUI)

Napari is “a fast multi-dimensional image viewer designed for browsing, annotating and analyzing large multi-dimensional images” (Napari Contributors, 2021). It is a well-implemented PyQT-based application framework with a broad user community, which provides an extensible base for building application-specific annotation and segmentation tools. Napari is encouraging the growth of an ecosystem of additional image processing plugins providing features such as animation (Sofroniew, 2021), tracking (Prigent, 2021), and deconvolution (Perdigao, 2021). By utilizing Napari as a common GUI, a user can access a range of tools from multiple authors but following similar conventions for improved ease-of-use and inter-operability.

A key benefit of an interactive GUI for performing image processing operations is the ability to interactively explore an image dataset. Napari provides a broad set of tools for 2D and 3D inspection of the image data, interactive painting of annotations, and visualization of outputs from each stage of a processing pipeline. Additionally, Napari supports overlaying multiple floating point image layers, each with controllable opacity, which can be helpful when viewing two or more registered images together. In addition, integer-valued annotation layers and geometry layers can be viewed together with floating point image layers allowing many different data types to be displayed concurrently for correlational painting of segmentations and labelling of objects. Using the 3D mode provides options for 3D rendering of data and segmented objects including isosurfaces and attenuated maximum intensity projection.

Napari and the SuRVoS2 plugin can run on Linux, Windows, and Macintosh operating systems (*see* https://github.com/DiamondLightSource/SuRVoS2 for code, installation instructions and documentation). This arrangement of SuRVoS2 as a Napari plugin both extends SuRVoS2 with access to 2D and 3D visualization and annotation tools that Napari offers and likewise, extends Napari by providing a complete system for managing segmentation and image analysis workflows, including an extensive set of filters, supervoxels, and shallow and deep machine learning segmentation options.

### Machine Learning Implementations

SuRVoS2 provides both a shallow and a deep machine learning pipeline (Figure 1). The shallow learning pipeline is equivalent to the functionality previously available in SuRVoS (Darrow et al., 2017; Luengo et al., 2017) whereby an initial set of training annotations are created and then an image segmentation model consisting of supervoxel-based image features and a random forest or SVM model is trained and evaluated, and then additional annotations or refinements of annotations are produced, and the model retrained and re-evaluated. The deep learning pipeline uses the 2D U-net from FastAI (Howard and Gugger, 2020) as a base and implements this functionality using The Kornia library (Riba et al., 2020) which was built using the Pytorch library (Paszke, et al., 2019), the Torch-IO library, some machine learning functions from scikit-learn (Pedregosa et al., 2011) and some image processing operations in SuRVoS2 also use the Scipy ndimage package (Virtanen et al., 2020) and the scikit-image library (van der Walt et al., 2014). These image processing technologies allow for GPU-accelerated computing to accommodate large datasets. Some additional image processing operations in SuRVoS2 use the Scipy ndimage package (Virtanen et al., 2020). The deep learning pipeline available in SuRVoS2 uses a region of interest (ROI) system whereby smaller, more manageable volumes are segmented either manually or through the shallow learning pipeline and used as training data. By providing one or more segmented ROIs as training data, the deep learning pipeline can transfer this learning to the rest of the volume. A multi-axis 2D U-Net (King, 2021) is used to predict the output segmentation in different directions, combining the output into a final segmentation. To enable faster processing, Dask is used for parallelized image processing of chunked data. Together these features of SuRVoS2 enable the efficient processing of large datasets.

### SuRVoS2 API

Complex segmentation problems usually require custom workflows. A Python API for SuRVoS2 can be accessed from either Jupyter notebooks (Kluyver et al., 2016), Python scripts or any other client capable of performing HTTP requests. A set of Jupyter-specific convenience functions allows for the inspection, creation, and modification of data within a SuRVoS2 workspace and give control of SuRVoS2 segmentation pipelines (*see* https://github.com/DiamondLightSource/SuRVoS2 for a testing notebook with examples). The output of these pipelines can be loaded into a common workspace for visualization and evaluation in the SuRVoS2 GUI. Additionally, SuRVoS2 is extensible by end users through a plugin mechanism composed of two parts: an API and a GUI. The API is implemented as a Python server using the Hug library (Crosley, 2016) to provide the REST API allowing for access to the workspace and all data in it. The GUI is a separate Python file consisting of the PyQT-based (RiverbankComputing.com, 2021) widgets that will be rendered in a tab within the SuRVoS2 user interface. For example, the SuRVoS2 ROI plugin has an API module that stores the ROI on the server and that can create and delete ROIs. Then it has a GUI module that contacts the API, gets the current list of ROIs, displays it, and allows the user to interactively create and delete ROIs.

## Results

SuRVoS2 implements multiple new features which accelerate image annotation, segmentation, and analysis. Example case studies have been sourced to highlight these features in the context of vEM and correlative imaging techniques. First, X-ray microCT of human placenta will be used to highlight the deep learning pipeline (Tun et al., 2021); second, cryo soft X-ray tomography (cryoSXT) of *Trypanosoma bruceii* will be used to demonstrate the label splitting and analysis functionalities; third, cryo electron tomography of a virus-infected cell will be used to illustrate the display and manipulation of non-mask based data such as the output from a distributed citizen science-based annotation workflow; fourth, correlative cryoSXT and cryo structured illumination microscopy datasets of a virus-infected cell will be used to demonstrate the ways correlative datasets can be used in the SuRVoS2 pipeline; and finally, an example Jupyter notebook will be used to highlight an advanced implementation of the SuRVoS2 API for clustering, segmentation, and visualization within the GUI.

### Deep Learning With the SuRVoS2 Implementation of a 2D U-Net

The two main segmentation methods available in SuRVoS2 can be used together to rapidly generate high quality segmentations for large datasets (Alvarez-Borges et al., 2021; Tun et al., 2021). SuRVoS2 implements a version of the concept of “weak annotation” in which scribble-based annotation is used to train a shallow machine learning model and then the prediction of that model is used as ground truth to train a deep learning U-Net (Lin D. et al., 2016; Khoreva et al., 2017; Li et al., 2018). This approach requires expert evaluation of the output predictions of both the shallow and deep machine learning models and is classified as a semi-automatic method. A subvolume (ROI) of the dataset is first selected, then supervoxels are generated to aid fast annotation of this ROI. This subvolume annotation can be done entirely manually, utilizing the benefits of supervoxels, or partial annotations, in conjunction with extracted image features, can be used to train an ensemble (e.g. a random forest) or an SVM classifier. The trained classifier is then used to predict the missing annotations within the ROI. After this step, the resulting segmented ROI and the corresponding data can then be used as “expert segmentation” data to train a U-Net network. This trained U-Net model is then available to use for predicting segmentation of the entire large dataset (*See* Tun et al., 2021 for validation of this methodology).

U-Net models have advantages over ensemble or SVM methods (Ronneberger et al., 2015; Seo et al., 2020) in terms of generalizing better to new data and in not requiring user-led extraction of image features for training and prediction. The U-Net, by contrast, learns the features to extract from the data during model training. However, deep learning models like the U-Net often require much larger amounts of training data to perform well. Therefore, the practical application of deep learning for segmentation suggests the use of a shallow machine learning pipeline, trained quickly using supervoxels, for the creation of training data that can be used as input to the deep learning pipeline.

**FIGURE 2:**
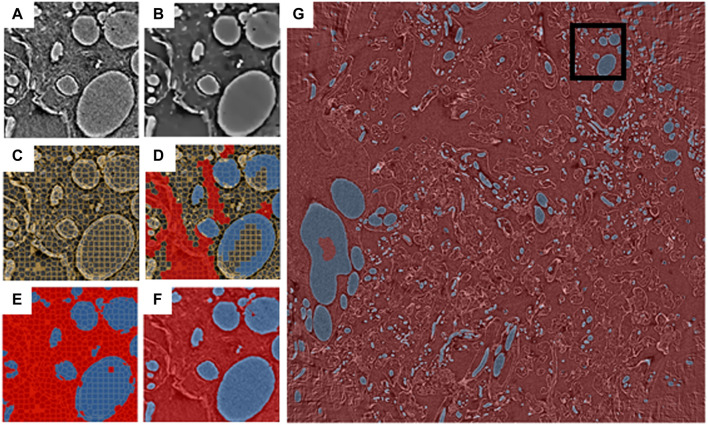
Blood vessels from an X-ray micro-tomography dataset of human placenta segmented using the SuRVoS2 deep learning pipeline. Images **(A**–**F)** show a central slice of a 256 × 256 × 256 pixel region of interest (ROI), marked in **(G)** with a black box. **(A)** Raw data. **(B)** Data with a total variation denoising filter applied. **(C)** Supervoxels generated from the denoised data. **(D)** Annotations applied to the supervoxels (red: background, blue: blood vessels). **(E)** Supervoxel-based prediction of segmentation labels using a random forest classifier trained on the annotations shown in **(D)**. **(F)** Voxel-based prediction of segmentation labels using a 2D U-Net model trained on the segmentation output from **(E)** and the raw data **(A)**. **(G)** Voxel-based predictions of segmentation labels for a central 100 slices of the full 2520 × 2520 dataset using the 2D U-Net model trained as described in **(F)**.

To demonstrate the above pipeline, an X-ray micro-tomography dataset (EMPIAR 10562, Tun et al., 2021) collected from a 3 × 3 × 3 mm sample of human placental tissue at Diamond Light Source beamline i13-2 was used (for more details of sample preparation and data collection *see* Tun et al., 2021). The full dataset has dimensions 2520 × 2520 × 2120 pixels and a 256 × 256 × 256 pixel ROI was selected from this dataset using the SuRVoS2 data previewer (Figure 2A). A workspace was created from the ROI and the data was denoised using a total variation filter (Figure 2B) before supervoxels with an average shape of 10 × 10 × 10 pixels were generated from this denoised volume (Figure 2C). A paintbrush tool was used to annotate some regions in the data (Figure 2D), separating the background supervoxels (shown in red) and those representing blood vessels (shown in blue). Further feature images were created from the ROI, namely a Gaussian blur, Hessian eigenvalues and normalized Gaussian datasets (not shown). The supervoxel annotations were used, along with the extracted image feature datasets, to train a random forest classifier which was then used to predict the labels for all of the supervoxels in the ROI (Figure 2E). At this point, the segmentation of the 256 × 256 × 256 pixel ROI is broadly correct with some minor misclassifications. This ROI segmentation was used to train a 2D U-Net model using an approach which leverages the 3D nature of the data (Alvarez-Borges et al., 2021). To do this, 2D images from the raw data and corresponding segmentation label volumes were sliced in three orthogonal planes to create the stack of training images. This process yielded 768 images of 256 × 256 pixels with the corresponding “ground truths.” These are enriched further by augmentations such as flips, contrast adjustments, and geometric distortions. Once trained, the process creates an output by taking the volume to be predicted and again dividing it into three stacks of 2D images, and augmenting this with rotations. This results in each voxel being classified multiple separate times and a voting system is used to select the final outcome of the voxel segmentation. The predicted segmentation of the ROI generated by this U-Net model has improved on the shallow learning segmentation output, addressing many of the misclassifications found there (Figure 2F).

**FIGURE 3:**
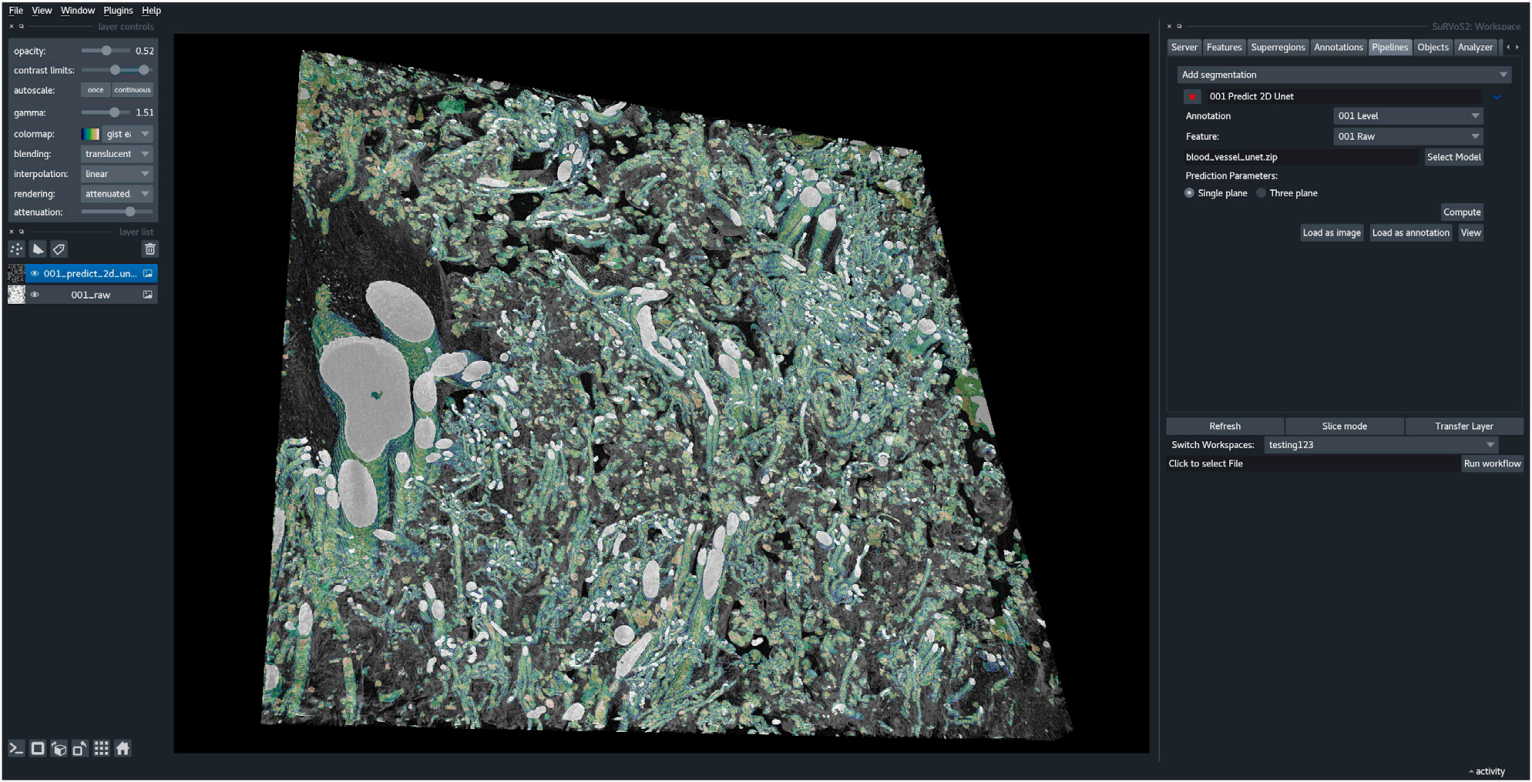
SuRVoS2 plugin integrated into the Napari viewer. The output blood vessel segmentations from the deep learning pipeline in SuRVoS2 visualized using the 3D rendering option and overlaid on the raw data in the Napari viewer.

Next, a new workspace was created containing a central portion of the full-size dataset (2520 × 2520 × 100 pixels). The U-Net model, trained as described above, was then used to generate segmentations from this larger volume of data (Figure 2G). Utilizing the capabilities of the Napari viewer and SuRVoS2 plugin, these segmentations can be rendered in 3D and overlaid with the image data to clearly visualize the now segmented blood vessels (Figure 3). Due to the large data size, manual segmentation of this single volume is estimated to have taken approximately 320 h, or around 2 months of person-time. Using the initial, shallow learning implementation of SuRVoS was estimated to have reduced the time spent by half, still taking approximately 1 month of person-time. Using the deep learning pipeline in SuRVoS2, segmentation of this volume was reduced to approximately 1.2 h of person-time to segment the ROI using the shallow learning pipeline and 4 h of computational time on a high performance machine consisting of two Intel^®^ Xeon^®^ Gold 6242R processors each with 20 cores running at 3.1 GHz, and 768 GiB of system memory. The GPU used was an NVIDIA Tesla V100 with 32 GB of available memory.

### Data Analysis in SuRVoS2

During segmentation, the class each object is assigned to is often left to the individual researcher based on 2D examination of the objects present in the data, leading to potential subjectivity in the results. The Label Splitter tool was developed as a means to derive classifications directly from the data using the inherent characteristics of each object, such as size, shape, intensity, variation, etc. Category labels and rules to generate classes are still decided by a user, meaning some bias or subjectivity is still present, however it will now be consistently applied, even across multiple normalized datasets.

**FIGURE 4:**
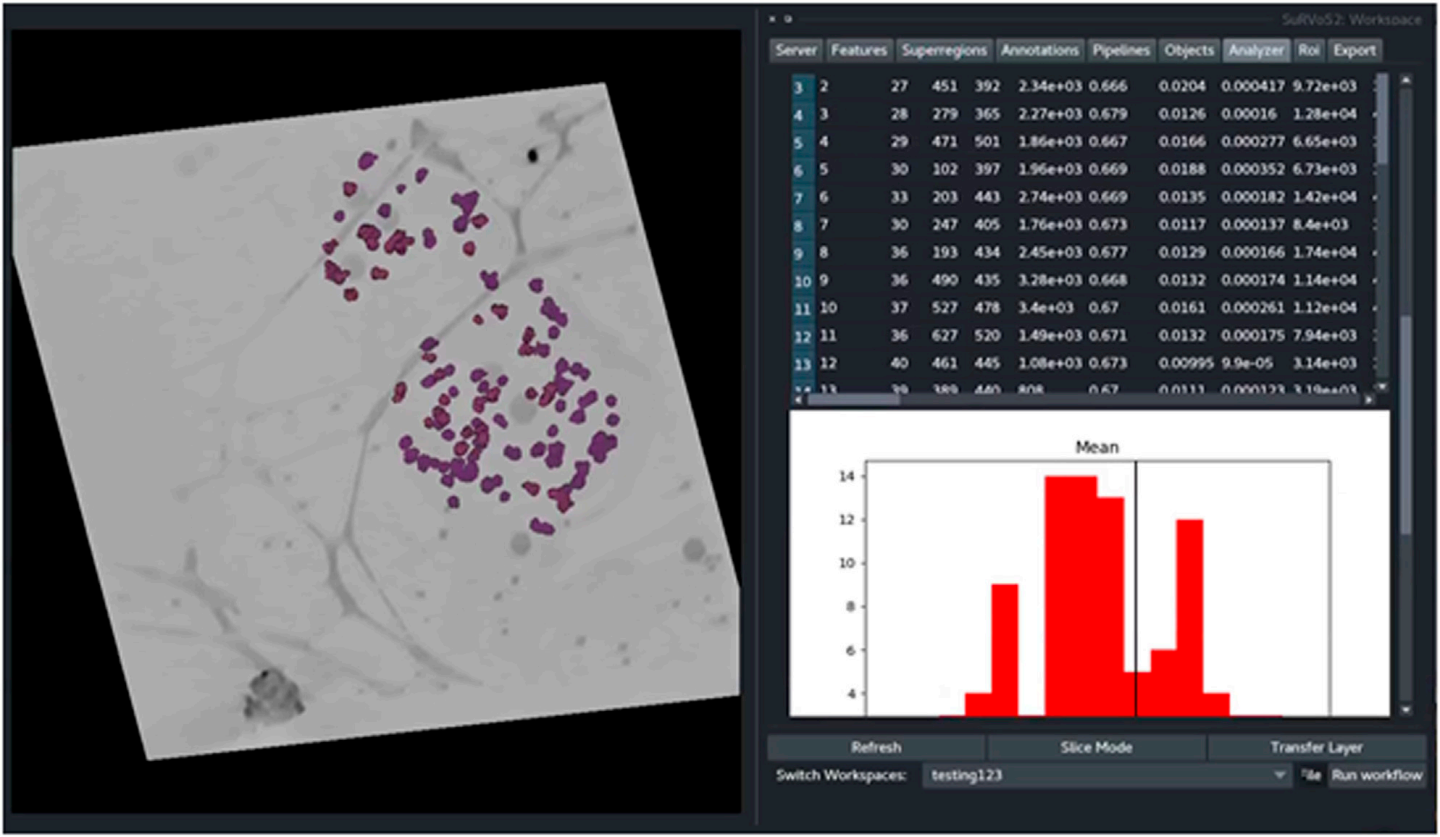
The Label Splitter tool for application of classification rules using inherent characteristics of the segmented objects. Information about each object is displayed on a table and graph. The graph is split by a line at the location of the chosen rule value and double clicking on an entry in the table takes the user to the chosen object in the data.

To demonstrate the functionality of the Label Splitter tool, a cryoSXT dataset of *Trypanosoma bruceii* collected from Diamond Light Source beamline B24 was used (*see* Darrow et al., JoVE, 2018 for more information). Starting from previously segmented objects, the Label Splitter tool displays information about each object that can be used to define rules for separating the objects into classes (Figure 4 and Table 1). These rules can be interactively developed using the table and graph view. The graph is split by a line at the location of the chosen rule value and double clicking on an entry in the table will take the user to the object in the data allowing for clear, specific delineations of classes of object based on the inherent characteristics of the objects.

**FIGURE 5:**
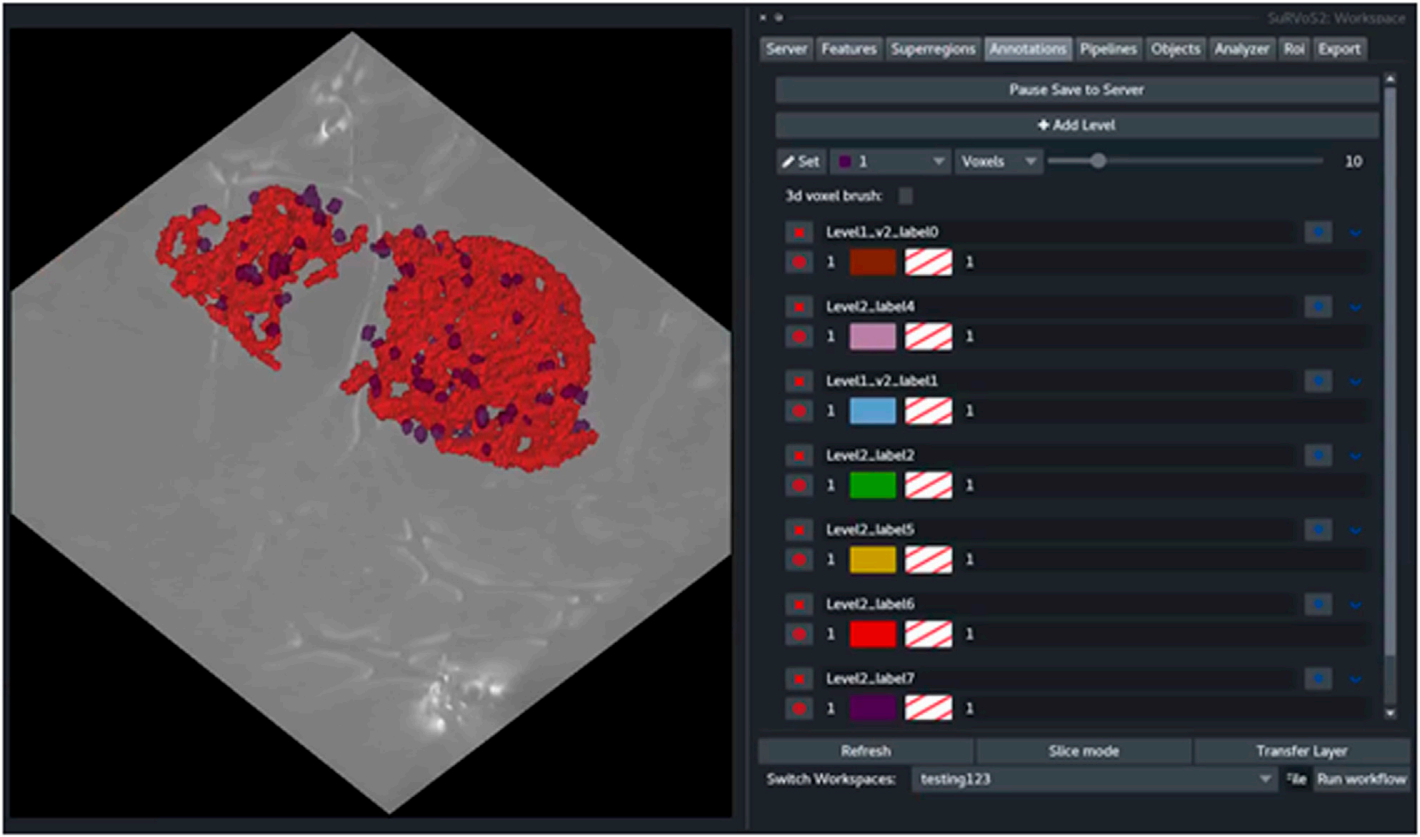
The output of the Label Splitter can be visualized as annotation layers within the SuRVoS2 plugin. Rules are applied sequentially to all selected objects and after splitting, each new class can be visualized and given a unique label and color.

Rules are applied sequentially to all selected objects creating bespoke classes of objects. After splitting, each new class can be visualized and given a unique label and color (Figure 5). The Label Splitter tool can be used on annotation layers regardless of their provenance (manual, shallow learning, deep learning or geometric) and it can also be used iteratively using the output of another Label Splitter instance to create complex and nested hierarchies that can accommodate data with large numbers of different types of objects.

### Geometric Data in Segmentation Workflows

SuRVoS2 supports segmentation projects that utilize geometric point data which can be visualized, edited, and used to generate mask-based annotations. Geometric data can be generated in many different ways, for example point-based locations of particles of interest in a 3D volume produced manually or automatically for sub-tomogram averaging (Wagner et al., 2019; Bepler et al., 2020); or through object detection workflows specific to an organelle or other object (Wei et al., 2020), or even points placed on images as part of distributed annotation workflows within a lab or utilizing citizen science platforms (Zooniverse, 2019).

Geometric data is an efficient way to encode knowledge about an image, where a point indicates both an object’s class membership and its location. To demonstrate the use of SuRVoS2 with geometric data, point-based data gathered from a crowdsourced workflow focused on finding viruses inside of a cell using cryo electron tomography (cryoET) data collected at Diamond Light Source on eBIC was used (Sutton et al., 2020)

**FIGURE 6:**
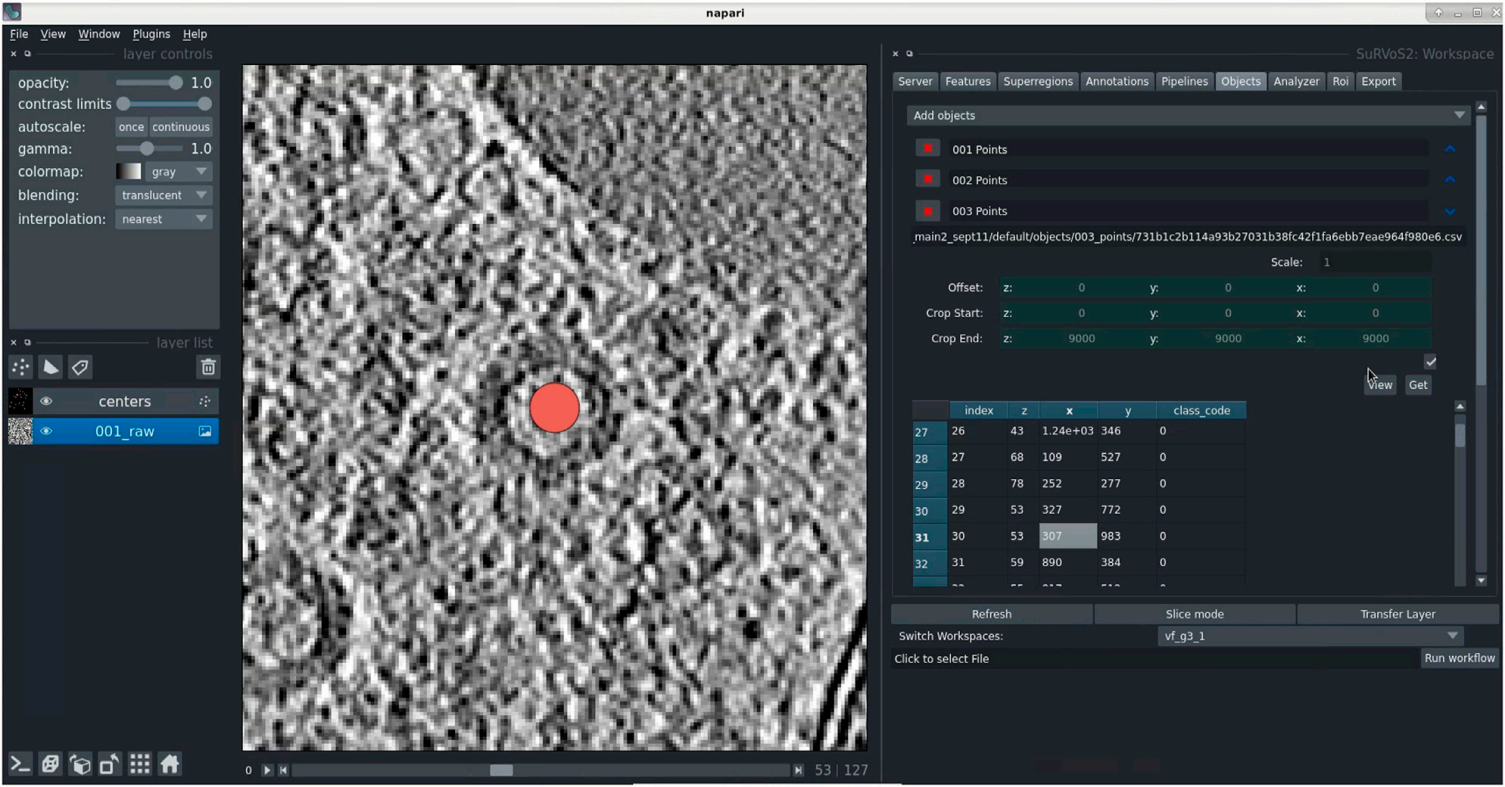
Use of geometric data in SuRVoS2 to analyze crowdsourced annotations. **(A)** Data were imported into the Napari viewer using a simple CSV file format indicating 3D centroid locations and object class. The tools in SuRVoS2 can be used to visualize, edit, and delete geometric data, including a function to take the user directly to an object of interest by double clicking on its entry in the table view.

It is possible to either create or import geometric data using Napari. In this case, the data were imported using a simple CSV file format. Points can then be viewed (2D and 3D) and edited/deleted (2D only) while overlaid with the raw image data as reference using the SuRVoS2 plugin (Figure 6). Point data can have an encoded class label represented by the point color. The table view lists the coordinates of the 3D point and its class. Double-clicking on the row of a particular point translates the current view to that location, centering the point (Figure 6). Together these capabilities provide users the tools needed to evaluate and refine crowdsourced annotations and those generated by automated object detection workflows (Jaeger, 2018).

The Rasterize Points plugin in SuRVoS2 can convert point-based data into segmentation masks by painting ellipsoidal blobs on the 3D point locations. The scale and orientation of the ellipsoids can be set and this operation allows the ellipsoid to be initialized as an Active Contour Without Edges (Chan and Vese, 2001) object which can expand or contract, fitting the image data. This or more complex conversion strategies for computationally converting point-based data into segmentations can be designed using the machine learning pipelines within SuRVoS2 (Figure 1).

### Extended Functionality Using the SuRVoS2 API

**FIGURE 7:**
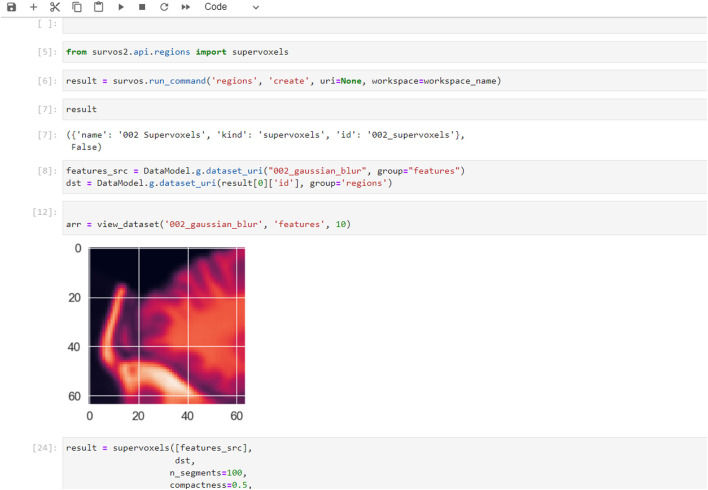
Examples of additional features and use-cases available when using the SuRVoS2 API from within Jupyter notebooks. Example of the SuRVoS2 API being used through a notebook with results which can be visualized using the SuRVoS2 GUI.

SuRVoS2 includes a Python API that can be used from Jupyter Notebooks, allowing the notebook to access, process, and add information into SuRVoS2 workspaces through the SuRVoS2 server (Figure 7). Additional functionality implemented through the API includes clustering and visualization of patches sampled from the volume. For example, a set of geometric points is used as sampling locations for uniformly sized patches (e.g. 64 × 64 × 64 pixels) and image features can be computed for each patch using a 2D ResNet model (He, et al., 2015). The features can then be clustered and an embedding made to visualize the features on a 2D plot using Unified Manifold Approximation and Prediction (UMAP; McInnes, et al., 2020) or TSNE (Van der Maaten and Hinton, 2008) (Figure 7).

As a second example also starting from geometric data with location and class information, the SuRVoS2 API can be used from any Jupyter client to segment the objects using additional deep learning strategies (such as a 3D U-Net or Feature Pyramid Network (FPN; Lin T et al., 2016) Figure 7). The training of the model is performed through the Jupyter notebook, a prediction is made, and the results are visualized as a layer in the SuRVoS2 GUI to allow for inspection of the segmentation. The ability to call standard and additional SuRVoS2 functionality external to the GUI extends the addressable use-cases and provides any researcher with Python programming skills the ability to create bespoke, specialized processing pipelines to address their specific annotation, segmentation, and analysis needs.

### Multimodal Correlation of 3D Datasets in SuRVoS2

**FIGURE 8:**
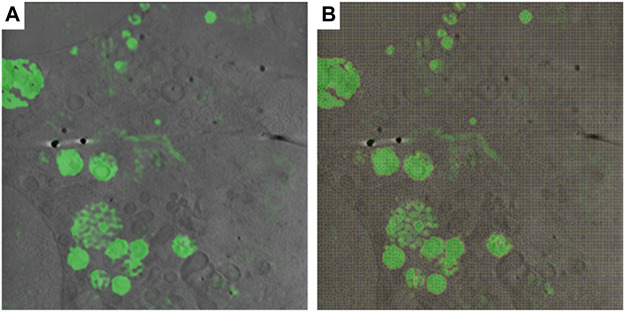
3D CryoSXT with correlated 3D cryoSIM in SuRVoS2. **(A)** Multimodal imaging can be displayed together as individual layers with control over opacity and color. **(B)** SuRVoS2 specific functionality, such as supervoxels (shown in yellow) can be calculated using one of the datasets for use during segmentation or annotation of the other.

SuRVoS2 supports the use of correlative, multi-modal imaging data for visualization and annotation. Multiple datasets can be loaded into a workspace and viewed in 2D or 3D as individual layers. The datasets must already be appropriately transformed as this functionality is not currently available in either Napari or SuRVoS2. To demonstrate the use of SuRVoS2 with correlative, multi-modal data, cryoSXT and cryo structured illumination microscopy (cryoSIM) of virus infected cells were aligned externally and displayed within SuRVoS2 (Figure 8; EMPIAR 10416 and S-BIAD19 respectively). The opacity of each layer can be controlled allowing for display of both 3D layers simultaneously.

This functionality allows the user to reference one dataset when annotating another dataset, for example using the fluorescence to guide segmentation of specific organelles. It is also possible to use other Napari and/or SuRVoS2 functionality to accelerate the annotation or segmentation process. For example, a deconvolution algorithm could be applied (e.g. RedLionfish (Perdigao, 2021) in Napari) to the fluorescence data, followed by calculation of supervoxels and then segmentation of areas of fluorescence in the non-fluorescence dataset. It is also possible to use the 3D fluorescence dataset as an input feature map during the shallow or deep learning pipelines or as a path to generation of point-based geometric data, although more research is needed to understand best-practices for these applications.

## Discussion

Segmentation is a difficult and time-consuming task that absorbs the attention and expertise of domain experts and software engineers alike. The general principles which have led to successful SuRVoS2 segmentations include using step-wise and hierarchical approaches. Rather than attempting to segment all objects in the field of view at once, a more successful strategy may be to segment large regions or distinctive objects from the background as a first step. This can then be followed by additional iterations through the segmentation pipelines, now utilizing the parent-child hierarchy to limit annotation and segmentation calculations to only regions where the objects of interest are to be found. By breaking the problem up into smaller chunks, the semi-automated computational strategies are generally more successful. It is also helpful during the expert annotation stage to be mindful of class imbalances. If an object of interest is only present a small number of times, or only represented by a small number of voxels in the training data, it will be difficult for the deep learning pipeline to accurately detect these objects in the remaining volume. Areas for expert annotation should be chosen carefully to ensure they represent the remaining volume, and secondary areas may be required to emphasize objects which do not occur often.

SuRVoS2 has several key strengths and provides a unique combination of tools that support this iterative process required for producing accurate output segmentations. First, SuRVoS2 encourages interactive visualization and exploration of image volumes and associated geometric data in both 2D and 3D within the user-friendly Napari environment. This is useful when understanding what objects are present in the data and choosing representative ROIs. Second, SuRVoS2 provides several different mechanisms to paint and prepare annotations, including supervoxels for accelerated annotation. And finally, SuRVoS2 provides both a fast mechanism for sparsely training a shallow model to predict annotations as well as a state-of-the-art deep U-Net segmentation model that can learn on densely annotated small ROIs and predict on large volumes.

SuRVoS2 core functionality is available entirely within the GUI for users who wish to interact without programming or scripting. Using the combination of tools available in SuRVoS2 to both annotate data and to train segmentation models, a domain expert can accelerate the process of segmenting their data and further can perform a variety of analyses from the output segmentations, all within the GUI. Additionally, for users familiar with scripting, the SuRVoS2 API and convenience functions allow for accessing and manipulating the entire process of segmentation within a SuRVoS2 workspace from within a Jupyter notebook. Importantly, the SuRVoS2 GUI can be run in parallel with Jupyter notebooks that are accessing the same workspace, providing an interactive analysis or development experience with access to 2D and 3D visualization options. Additionally, application developers can utilize the SuRVoS2 plugin system to extend SuRVoS2 functionality with their own custom plugins. This growing ecosystem within SuRVoS2 and Napari provides an exciting outlook for the future of volumetric image analysis and visualisation.

By separating SuRVoS2 into a client-server architecture, the hardware requirements and installation challenges of the software have been alleviated. The client, installed on a personal or work computer, requires minimal effort to install; and the server installation, which has also been simplified, can be completed by expert technicians on clusters and/or in the cloud to serve a large user base.

### Future Development Plans

Now that the client-server architecture has been implemented, the immediate bottleneck associated with processing large datasets within SuRVoS2 has been alleviated, especially when the ROI-based deep learning pipeline is used. However, more efficient use of Dask cluster can be implemented alongside the use of Next Generation File Formats (Moore et al., 2020) to allow further parallelization of image processing operations which will allow SuRVoS2 to scale to even larger datasets.

A second area of development is around use of SuRVoS2 in the cloud. This has been tested with a limited set of virtual machines (Google cloud with Chrome Remote Desktop for client access). This configuration has provided good performance without loss of interactivity. In future, this will be supported through use of a SuRVoS2 server docker container, which could be hosted in the cloud and connected to an external client. This will also enable the possibility of using mobile and browser-based clients for further improvements to accessibility.

Third, SuRVoS2 will benefit from updates and improvements provided by the Napari plugin ecosystem. Napari provides a common user interface paradigm for all Napari plugins to use, easing the burden on researchers to learn bespoke plugins. And the functionality provided by new Napari plugins, especially in the area of segmentation tools, for example Stardist (Schmidt, et al., 2018) and ZELDA (D’Antuono and Pisignano, 2021) can be incorporated within a SuRVoS2 workflow.

A final area of research and development within the SuRVoS2 team is around the need for quantitative quality metrics embedded within the annotation and segmentation process. Segmentation of volumetric biomedical data using machine learning strategies often requires multiple iterative passes of annotation, segmentation, and quantitative evaluation to achieve high-quality results. It is often difficult or impossible to determine how much annotation is required and how detailed the annotation must be to achieve a particular segmentation result. Evaluation of the segmentation output is often manual and subjective and often completed in 2D, highlighting the need for quantitative metrics embedded within the segmentation pipeline to enable the evaluation of the segmentation output. In the future, we plan to explore active learning and collaborative learning techniques (Konyushkova et al., 2015; Luengo et al., 2016; Yang et al., 2017), which will aid the user in selecting regions for annotation to reduce model uncertainty. This deep learning aided segmentation strategy has the potential to further reduce the amount of manually annotated data through smarter selection of ROIs.

## Conclusion

SuRVoS2 offers a unique set of tools that combine image processing and segmentation with the management of geometric information and the tools available in the Napari viewer. Its redesigned client-server architecture and newly implemented deep learning segmentation pipeline addresses the need to process ever larger datasets. The advanced features provided by the SuRVoS2 API allow users comfortable with scripting to interact through a Jupyter notebook while still accessing the interactive viewing tools. This ecosystem is designed for extensibility through plugin systems both within Napari and SuRVoS2. Together, these features allow for easy exploration of volumetric biomedical and correlative multi-modal data in both 2D and 3D followed by accelerated segmentation pipelines and analysis tools, accelerating data processing and analysis across modalities and scales.

## Data Availability

Publicly available datasets were analyzed in this study. This data can be found here: EMPIAR 10562: https://www.ebi.ac.uk/empiar/EMPIAR-10562/ EMPIAR 10416: https://www.ebi.ac.uk/empiar/EMPIAR-10416/ S-BIAD19 (bioimage archive): https://www.ebi.ac.uk/biostudies/BioImages/studies/S-BIAD19?query=S-BIAD19 SuRVoS2 GitHub: https://github.com/DiamondLightSource/SuRVoS2.
