# Supplementary material for: SuRVoS 2: Accelerating Annotation and Segmentation for Large Volumetric Bioimage Workflows Across Modalities and Scales
Source: Front Cell Dev Biol. 2022 Apr 1;10:842342. doi: 10.3389/fcell.2022.842342 (PMC9011330; doi:10.3389/fcell.2022.842342)
Supplement: Supplementary file 1 [file DataSheet1.PDF]

Filters & Features	Machine Learning	Label Splitter/Analysis
Produce output images for use in shallow or deep learning pipelines	Uses manual or shallow learning created training annotations to predict classes for other voxels	Inherent characteristics of data or segmented objects used to separate objects into groups
Basic Features:	Shallow Learning:	Mean Intensity
Simple Invert	Random Forest	Standard Deviation of Intensity
Invert Threshold	Extra Random Forest	Variation of Intensity
Threshold	Gradient Boosting	Volume
Rescale	Support Vector Machine (SVM)	Bounding Box Volume
Gamma Correct	Active Contour without Edges (ACWE)	Log Bounding Box Volume
Blob:	Watershed	Position X
Structure Tensor Determinant	Deep Learning:	Position Y
Frangi	2D U-Net	Position Z
Hessian Eigenvalues	3D U-Net *	Bounding Box Depth
Denoising:	FPN *	Bounding Box Height
Total Variation Denoise		Bounding Box Width
Gaussian Blur		Oriented Bounding Box Volume
Median		Log Oriented Bounding Box Volume
Wavelet		Oriented Bounding Box Depth
Edges:		Oriented Bounding Box Height
Spatial Gradient 3D		Oriented Bounding Box Width
Difference of Gaussians		
Laplacian		
Morphology:		
Dilation		
Erosion		
Closing		
Euclidean Distance Transform		
Skeletonize		
Neighborhood:		
Gaussian Norm		
Gaussian Centre		

* Training is done through the SuRVoS2 API, but the deep learning module for prediction is available within the SuRVoS2 GUI.
